# Communication dynamics and media interactions of young adults who have attempted suicide: a qualitative thematic analysis

**DOI:** 10.3389/fpsyg.2024.1460348

**Published:** 2024-11-01

**Authors:** Emel Arık, Mustafa İnce, Mevlüt Can Koçak, Yasemin Bilişli, Emrah Onur Karataş, Hakkı Akgün, Faruk Aşlakçı

**Affiliations:** ^1^Department of Journalism, Faculty of Communication, Akdeniz University, Antalya, Türkiye; ^2^Department of Journalism, Turker Inanoglu Faculty of Communication, Karabuk University, Karabuk, Türkiye; ^3^Department of Radio, Television and Cinema, Turker Inanoglu Faculty of Communication, Karabuk University, Karabuk, Türkiye; ^4^Department of Office Services and Secretariat, Social Sciences Vocational School, Akdeniz University, Antalya, Türkiye; ^5^Department of Journalism, Faculty of Communication, Suleyman Demirel University, Isparta, Türkiye

**Keywords:** communication dynamics, digital media usage, media interaction, qualitative research, suicidal tendencies, suicide attempt, thematic analysis, young adults

## Abstract

**Objective:**

The study examines the potential effects of communication processes and media consumption habits on suicide ideation among male and female young adults aged 18–29 who have attempted suicide at least once.

**Methods:**

In-depth interviews were analyzed using MAXQDA Analytics Pro 2024, and thematic analysis was applied according to Braun and Clarke’s model.

**Results:**

Four themes emerged: (1) Family-related factors, (2) sociopsychological factors, (3) sociocultural factors, and (4) media-related factors. Regarding family-related factors, most participants come from broken family structures and commonly report issues with family communication and experiences of violence. Regarding sociopsychological factors, anger issues, despair, and addictions among participants were observed to increase suicide tendencies. Regarding sociocultural factors, most participants expressed difficulties in conforming to society and feeling pressures from cultural or religious expectations. Regarding media-related factors, it was noted that a vast majority of participants spend long hours consuming media daily and frequently interact with content that leads them into adverse emotional states, primarily for time passing on social media platforms.

**Discussion:**

This research not only reinforces information in the literature but also presents unique findings compared to similar studies, particularly in cultural and geographical contexts. The results uniquely highlight the diversity in perceptions of the relationship between religion and suicide. While literature generally notes religion as a deterrent to suicide, this study reveals that intense religious pressure could increase suicidal tendencies through effects like rejection and hatred of religious values. Media also plays a reinforcing role in this context.

**Conclusion:**

In conclusion, this study elucidates the complex interactions underlying suicide attempts among young adults and provides a solid foundation for policies and interventions aimed at better managing media interactions, which play a critical role in suicide prevention efforts.

## 1 Introduction

Suicide can be defined as the act of intentionally killing oneself. In other words, suicidal behavior includes attempts, threats, thoughts, and actions that result in self-harm (World Health Organization, 2024). Although these actions are not classified as a disease, behaviors leading to suicide attempts constitute a significant public health issue (Turecki et al., 2019). This public health problem seriously affects many families worldwide (Ortiz-Sánchez et al., 2023). According to data from the World Health Organization (2024), more than 700,000 people die from suicide each year, with an estimated 20 suicide attempts for every completed suicide. In 2019, suicide was the fourth leading cause of death among young people aged 15–29 worldwide. Risk factors for suicide include experiences of loss, loneliness, discrimination, relationship breakdowns, financial problems, chronic pain and illness, violence, abuse, and conflict or other humanitarian emergencies (World Health Organization, 2023). Many factors play a role in the development of suicide risk, including biological (such as genetic factors), psychological, social, and environmental influences (Turecki et al., 2019). In other words, suicidal behavior is associated with personal, familial, and social factors.

Suicide can be considered a complex phenomenon with multiple dimensions. Therefore, it is not feasible to analyze or understand suicides that occur due to a single reason alone. All changes and transformations within the societal structure affect the phenomenon of suicide, leading to variations in its incidence. With this characteristic, the phenomenon of suicide is studied by different disciplines, seeking solutions (Diktaş Yerli, 2023). When approached as a multidisciplinary phenomenon, suicide can greatly benefit from the perspectives of various disciplines. Communication science, which examines communication processes, the use of language and symbols, communication media, and the social dimensions of communication, can provide significant contributions to understanding and analyzing the phenomenon of suicide. This study, which takes into account all the communicational dynamics of individuals who have attempted suicide, also encompasses data on media usage behavior and interactions, underscoring the importance of the information it will provide.

The study examines the potential effects of communication processes and media consumption habits on suicide ideation among male and female young adults aged 18–29 who have attempted suicide at least once. Therefore, answers to the following research questions will be sought throughout the study:

*RQ1*: What is the relationship between different family structures, communication styles, and suicide attempts?

*RQ2*: How do personality traits and psychological disorders influence the risk of suicide?

*RQ3*: How do societal communication patterns and cultural norms affect the risk and prevalence of suicide?

*RQ4*: In what ways do media usage habits, social media interactions, and content consumption influence suicidal tendencies?

By addressing the stated questions, this study may provide a comprehensive understanding of the personal, social, and media-related factors that contribute to suicidal behavior.

According to data from the Turkish Statistical Institute for 2022, a total of 4,061 individuals committed suicide in Türkiye in 2022. The distribution of suicides by age group indicates that the highest number of cases occurred in the 15–19 age group with 387 individuals, the 20–24 age group with 546 individuals, and the 25–30 age group with 554 individuals. These age groups constitute 36% of the total suicide cases (Turkish Statistical Institute, 2022). This data highlights that suicide rates among young adults are a significant concern. Additionally, according to TÜİK’s Household Information Technology Usage Survey, 95.5% of individuals in Türkiye have used the internet in the last 3 months. The survey notes that internet usage rates are higher in the 16–24 and 25–34 age groups. The most widely used social media platforms among participants are as follows: WhatsApp at 84.9%, YouTube at 69%, Instagram at 61.4%, Facebook at 51.4%, Twitter at 23.7%, and TikTok at 17.1% (Turkish Statistical Institute, 2023). Thus, young adults aged 18–29, who are both at high risk for suicide and are heavy internet users, have been chosen as the sample for this study.

The literature predominantly features quantitative studies. However, this research will obtain qualitative data through in-depth interviews with the target population, providing a more detailed exploration of individual experiences related to suicide tendencies and their relationship with media.

### 1.1 Literature review

Suicide is a complex phenomenon influenced by a wide range of personal, familial, societal, and environmental factors. In the existing literature, researchers have explored how these factors interact to shape suicidal tendencies, especially among vulnerable populations like adolescents and young adults. Among these factors, family dynamics, socio-psychological traits, and cultural norms play crucial roles in either exacerbating or mitigating suicidal behavior. Additionally, media influences, particularly through social media, have become increasingly relevant in the modern context. This section reviews key studies that examine these dimensions and their relationship to suicidal behavior.

Family relationships can significantly influence the suicidal behaviors of adolescents. Particularly, high levels of conflict, mistreatment, and violence are known to produce negative outcomes. Situations such as parental divorce, separation, and the use of alcohol and substances also have a strong association with suicidal actions (Wagner et al., 2003). Parental separations can affect the likelihood of contemplating and attempting suicide. A study in Sweden found that experiencing parental separation/divorce during childhood significantly impacts the presence of suicidal thoughts. This effect is notably higher in parental separations occurring between the ages of 0–4 (Lindström and Rosvall, 2015).

Victims of domestic violence are known to be at high risk for suicidal thoughts and attempts. Various studies have demonstrated the level of this risk. In Victoria, Australia, a study was conducted on cases of interpersonal violence exposure among people who died by suicide. For this study, 2,153 suicide cases between 2009 and 2021 were examined. The research found that 42% of the women who died by suicide had been exposed to interpersonal violence. Furthermore, while 23% of these women were subjected to physical violence, 18% experienced psychological violence, and 16% were subjected to sexual abuse. It was also found that a significant portion of men who died by suicide had been exposed to interpersonal violence before their death (MacIsaac et al., 2018). Similarly, suicide actions can increase in situations of economic uncertainty (Tolga et al., 2023). Economic violence and hardships can lead to a prevalence of depression in people (Marcotte and Hansen, 2024), which in turn can lead to suicidal actions.

Early childhood sexual abuse can be a risk factor for suicide. Research supports this assertion. In the United States, a study by Molnar et al. found that the likelihood of attempting suicide among those who had experienced sexual abuse in childhood was 4–11 times higher for men and 2–4 times higher for women compared to those who had not been abused (Molnar et al., 2001). According to Brent and Mann (2005), sexual abuse is also a risk factor for suicide. Different potential mechanisms can increase the contagion risk of suicidal behavior. Suicidal behaviors can be familial and may be hereditary due to at least two components. The first is psychiatric disorders, and the second is a predisposition to impulsive aggression. A predisposition to psychiatric disorders combined with a predisposition to impulsive aggression results in the highest risk for suicide. Particularly, the presence of impulsive aggression in a parent can also set the stage for domestic abuse, thereby increasing the risk of suicidal behavior in children (Brent and Mann, 2005). In their research, Afifi et al. (2009) found that when parental divorce accompanies child abuse, the likelihood of a suicide attempt is much higher compared to cases of child abuse alone (Afifi et al., 2009). Children at risk of attempting suicide include those with separated parents, those with weak parent–child interactions, and those who have suffered sexual abuse. Early exposure to such events, combined with depression and anxiety disorders, can trigger suicidal behavior (Fergusson et al., 2000).

In the realm of youth suicides, several risk factors emerge as particularly significant, including mental illness and family histories of suicide (Agerbo et al., 2002). Family histories of suicide are especially noteworthy (Brent and Melhem, 2008). A study by Hawton and colleagues supports this, finding that 35.6% of 146 patients had a known family history of suicidal behavior (Hawton et al., 2002). The presence of a family member who has previously attempted suicide—whether a sibling or another relative—significantly increases the risk for children (Brent and Mann, 2005). Family communication plays a crucial role in preventing suicides among family members prone to such actions. Conversely, suicides can adversely affect other family members. Research by McLaughlin et al. (2014) on families of individuals prone to suicide found that family members spend prolonged periods with the individual out of concern for repeated suicide attempts. Suicide not only harms the individuals’ own lives but also negatively impacts their close circles (McLaughlin et al., 2014). Families who lose loved ones to suicide can experience feelings of despair, guilt, and anger. Additionally, these families may face societal stigma (Lee, 2022). Suicidal actions can create a “climate of fear” among other family members. The fear that other suicide attempts may occur within the family can negatively impact their lives (Creuzé et al., 2022). Research by Rubenstein et al. (1989) on high school students who are and are not prone to suicide found that family cohesion balances the impact of stress. Adolescents who perceive their families as highly harmonious are significantly less likely to be prone to suicide compared to those who see their families as discordant and disconnected (Rubenstein et al., 1989).

Suicide acts are a significant cause of death among youth (Arango et al., 2024; Xuan, 2023; Hill et al., 2023). Particularly, three factors are considered major risk elements: personality traits, psychological factors, and stressful life conditions. Experiences of abuse or mistreatment in childhood and stressful living conditions can lead to a higher risk of suicide (Caro-Cañizares et al., 2024). Specifically, women who have previously been exposed to physical and sexual abuse are at a higher risk of developing mental disorders, which can make suicide actions more likely (Armoon et al., 2024).

Personality traits, which describe an individual’s characteristic behaviors, thoughts, and emotional attributes, often guide their responses. While personality traits can influence suicide risk, they are not solely responsible for causing suicide. Various studies have shown that anger and aggressive behaviors can be precursors to a propensity for suicide (Dillon et al., 2020; Hill et al., 2020). Additionally, factors such as hopelessness, helplessness, and lack of self-confidence have been identified as significant elements in individuals’ tendencies toward suicide, as seen in various studies (Brott and Veilleux, 2024; Liu et al., 2020; Grigienė et al., 2022).

Negative personal traits such as anger and aggressive behaviors, hopelessness and helplessness, low self-esteem, and poor communication not only increase tendencies toward suicide but also adversely affect substance use and involvement in crime (Toumbourou and Gregg, 2002). Alcohol use and suicide actions among youth are serious public health issues. Impulsivity and aggression can be high among those who attempt suicide. Disorders like alcohol and substance use disorders, antisocial personality disorders, impulse control disorders, and behavioral disorders can also be linked to suicidal behavior (Carballo et al., 2006). Similarly, drug use is considered a significant risk factor for suicidal behavior. Drug use can increase suicidal thoughts (Amiri and Behnezhad, 2020; Devin et al., 2023). Situations involving both substance use and social fragmentation can significantly increase the likelihood of death by suicide (Hunter et al., 2023). The connection between suicidal thoughts and substance use appears more frequently among university students. There is also a relationship between suicidal tendencies and alcohol use. Those who consume alcohol are at higher risk for suicidal behavior compared to non-users. Therefore, early intervention in tobacco and alcohol use among students is important for preventing suicidal thoughts and attempts (Wang et al., 2023; Conner et al., 2014; Giesbrecht et al., 2024). The rate of suicide is much higher among young and middle-aged adult men who consume alcohol (Yeskendir et al., 2023).

There is extensive research demonstrating the relationship between an individual’s psychological health and suicidal actions. One such study conducted by Bae et al. (2022) in South Korea found that 87% of those who died by suicide had mental health issues (Bae et al., 2022). It was also discovered that a significant portion of those who committed suicide had given warning signs prior to the act (Fernando et al., 2022). One of these warning signs was these individuals reaching out to mental health services. In other words, those contemplating suicide often contact health services before carrying out the act. These consultations can serve as indicators of suicide risk, thus providing an opportunity for intervention before a suicide attempt occurs. For example, if an individual has major depressive disorder, they may be more prone to attempt suicide (Feng et al., 2023; Yang et al., 2023).

Sociocultural factors are significant elements that influence suicide actions. Suicide typically emerges as a result of complex and multiple causes and is affected by the convergence of social, cultural, economic, and psychological factors. The societal system in which a person lives and relies upon not only empowers them with values and ideas but also ensures they are part of a balanced network of relationships. When this balance is not achieved, individuals may face problems maintaining their integrity (Fromm, 2017). For instance, looking at the relationship between suicide and religious ideology, it is evident that individuals join groups for religious reasons and that religious ideology primarily influences behaviors. Accordingly, there are differences in the relationships between suicide and religious ideologies across different beliefs. In Catholic societies, stronger social control and tighter family bonds provide significant psychological support and act as a deterrent to suicide. Thus, a high sense of belonging fosters a deeper understanding of life that prevents self-destruction (Zirojević and Marković, 2020).

The reasons for suicide actions can range from a loss of the will to live to sometimes creating social excitement or the notion of becoming a hero for a cause. Suicide can result from both positive and negative acquisitions (Durkheim, 2013). In other words, the absence of any action toward life can also be a form of suicide (Küçük et al., 2022). Often, the underlying reason for many suicides is the individual’s sense of isolation. An isolated individual “cannot establish a fundamental unity with the world” (Stellino, 2020). Adler (2024) states that people who choose individual lives are those who are distant from collaborative living and have chosen this life themselves. After this process, they may lack social interest and may attempt suicide because they see themselves as failures (Adler, 2024). As much as loneliness underlies many societal ailments, people often cite loneliness as a reason for suicide (Durkheim, 2013). Those who attempt suicide might do so believing their lives have been unsuccessful and are no longer worth continuing. In a sense, they think that by committing suicide, their failures will end (Fromm, 2017). People who feel defeated in life, who have faced disappointments and shattered dreams, may choose suicide. They believe that by committing suicide, they will be freed from their internal pain (Schopenhauer, 2013). Kirmayer’s study (2022) highlights the role of social factors and an individual’s communication with society in suicidal thoughts (Kirmayer, 2022). Another study on suicide found that social ostracism plays a significant role in suicidal actions (Wiglesworth et al., 2022).

Suicide can be considered a complex phenomenon with multiple dimensions. Therefore, it is not feasible to analyze or understand suicides that occur due to a single reason alone. All changes and transformations within the societal structure affect the phenomenon of suicide, leading to variations in its incidence. With this characteristic, the phenomenon of suicide is studied by different disciplines, seeking solutions (Diktaş Yerli, 2023). When approached as a multidisciplinary phenomenon, suicide can greatly benefit from the perspectives of various disciplines. Communication science, which examines communication processes, the use of language and symbols, communication media, and the social dimensions of communication, can provide significant contributions to understanding and analyzing the phenomenon of suicide. This study, which takes into account all the communicational dynamics of individuals who have attempted suicide, also encompasses data on media usage behavior and interactions, underscoring the importance of the information it will provide.

It is known that specific ways of reporting suicide in the media can increase suicide rates (Niederkrotenthaler et al., 2020). Research indicates that the more frequently suicides are reported in the media, the greater the increase in suicide rates (Pirkis et al., 2018). In Hong Kong, an increase in suicide cases among men aged 25–39 has shown the presence of a copycat effect in the media (Schmidtke and Schaller, 2000). The presentation of suicide in media reports can particularly affect imitative actions. Thus, there is a consistent relationship between media and suicidal behavior. Case studies have shown that reading news about suicide on internet media or joining social media groups that share content related to this topic can influence individuals prone to suicide (Pirkis et al., 2018). A study examining the relationship between social media and suicidal behaviors among youths in Ohio found that exposure to suicide content on social media significantly increased suicidal thoughts (Swedo et al., 2021). Similarly, a study conducted in Türkiye on individuals aged 18 and over has found that over 70% of participants specifically read news about suicide on the internet and social media. Another finding from the study is that more than 30% of the participants had previously considered suicide (Küçük et al., 2022).

Technology leads to the easy clustering of suicides among young individuals who heavily use social media, facilitating the spread of this idea through their peer groups (Massing-Schaffer and Nesi, 2020). Particularly for adolescents, social media has become a tool for recognizing and understanding what they see. A feeling of being misunderstood in offline environments has strengthened the impulse to interact more online, helping to normalize suicidal thoughts among adolescents and making them feel less alone in their struggles (Balt et al., 2023). The Coronavirus pandemic in 2020, which deeply affected the entire world, triggered depression, stress, and loneliness. Public health messages in the media, such as “stay home” and “social distancing,” have increased the sense of isolation and loneliness, particularly making young individuals more prone to suicide (Czeisler, 2020). A study in China highlights the importance of social communication for individuals prone to suicide and notes that social media platforms can effectively be used in preventative measures for individuals dealing with social integration and isolation issues, who are contemplating suicide (Cheng et al., 2015).

A scoping review found that social media has both positive and negative effects on suicidal thoughts. Since the advent of social media, individuals have increasingly used online platforms to express their suicidal tendencies (Malhotra and Jindal, 2022). People often tend to open up on social media platforms while concealing their identities, where many texts related to suicide can be found. From this point, social media as an open forum can provide great convenience for researchers who seek to view the “digital footprint” (Teo and Fu, 2021). It has been observed that young people who self-harm frequently use social media to express their distress and numerous cases have been identified where individuals have committed suicide after posting on social media. Furthermore, according to research, expressing suicidal intent through social media platforms can be seen as an unconventional way of seeking help. This situation has encouraged researchers to leverage the power of social media to prevent suicides. Thus, preventing suicides might be feasible by monitoring social media posts and analyzing online behavior, leading us back to social media platforms, which can be used to detect depression and similar health issues (Wongkoblap et al., 2017). Producing quality content in the media to raise awareness about suicide prevention and formulating programs and strategies is crucial (Kirchner and Niederkrotenthaler, 2024). Determining beliefs and attitudes related to suicide has become possible through social media. Identifying an individual’s attitudes on social media and the way technology and social media are used plays a key role in reducing suicidal tendencies and developing interventions (Keating and Rudd-Arieta, 2021).

The literature review has comprehensively addressed research on the communication dynamics and media usage behaviors of individuals prone to suicide but has also highlighted some significant gaps. Primarily, most existing studies focus on Western countries, and there is a noticeable lack of research in different cultural and geographical contexts. There are limited studies on the media usage behaviors of individuals who have attempted suicide and how these behaviors vary across different sociodemographic and cultural contexts. Moreover, detailed analyses of the media consumption habits of these individuals and the effects of these habits on their psychological states are lacking. This research aims to contribute to the existing literature by examining suicide tendencies and the role of media in different cultural and geographical contexts. The study conducted in the context of Türkiye will provide significant insights into suicide tendencies and the role of media in countries outside the West.

## 2 Materials and methods

### 2.1 Study design

The aim of this study is to qualitatively examine all communication dynamics and media interactions of young adults who have attempted suicide. The research focuses on the impacts of personal, familial, and socio-cultural factors on suicidal thoughts and behaviors, while emphasizing the effects of media consumption habits, social media usage, and media content on mental health. Data for the study was collected through in-depth interviews with 13 participants, based on a semi-structured guide. In qualitative research, sample size is often determined by the concept of data saturation, which is reached when no new themes or insights emerge from the data (Braun and Clarke, 2006). Studies with homogeneous groups and specific objectives, such as ours, often achieve saturation with sample sizes between 9 and 17 participants (Hennink and Kaiser, 2021). Given that our study focuses on young adults aged 18–29, a relatively homogeneous group, the sample size of 13 participants is consistent with these guidelines for achieving thematic saturation. After transcription, the research material was analyzed using MAXQDA Analytics Pro 2024 software. Thematic analysis began with the creation of initial codes from raw data, followed by stages of theme development to capture core patterns. This approach ensured the derivation of inductive insights from the data and alignment with existing theoretical frameworks. Thematic analysis has facilitated the identification of critical factors influencing suicidal tendencies and the role of media.

The interview guide was specifically designed for this study by the research team, with the support of two different academics specialized in the field of psychology (IK and AI), and based on the literature. The guide contains a list of open-ended questions to be addressed during the interviews. The questions cover topics such as family structure and cultural characteristics, habits, internet and media usage, psychosocial traits, influential individuals, societal perceptions, reasons behind suicidal tendencies, and the search for solutions. This guide was pilot-tested on 3 different participants before the main study. These interviews were not included in the study.

### 2.2 Setting

This study used two main inclusion and exclusion criteria. Young adults aged 18–29 who had previously attempted suicide at least once were included. However, those who had attempted suicide but were currently diagnosed with an acute psychiatric condition were excluded to avoid compromising the validity of the research findings. The interviews were conducted between February 1, 2024, and May 15, 2024. All interviews were carried out face-to-face in locations such as the participants’ homes or cafeterias, which they described as safe and comfortable. The interviews were recorded by the interviewers and then transcribed. The transcripts were not returned to the participants for comments, and the interviews were not repeated. No feedback on the findings was requested from the participants.

### 2.3 Data collection

The study was guided by the Consolidated Criteria for Reporting Qualitative Research COREQ (Tong et al., 2007). EA and Mİ designed the study and supervised its implementation. The interviews were conducted by MCK, FA, EA, YB, HA, and Mİ. The entire interviewing team consisted of academics from communication faculties in Türkiye. All interviewers had previous experience in conducting various qualitative studies and some researchers from the team had previously worked on topics related to the research subject. Research participants were informed in advance about what the interviewers aimed to achieve with this study and about their personal goals. Field notes were taken during and after the interviews. The interviews, lasting an average of 30 min (ranging from 20 to 60 min), were digitally recorded and then professionally transcribed verbatim. The interviews were translated into English and coded as participants (P1, P2…). During the translation, textual quotations were initially translated word-for-word and later adjusted to achieve equivalence in meaning and interpretation. EA and YB conducted the coding and thematic analysis. EOK performed the data analysis of the study using MAXQDA data analysis software. Information on how each of the 32 items of COREQ was addressed is provided in the Supplementary Table S1.

### 2.4 Data analysis and trustworthiness

In analyzing the data, Braun and Clarke’s six-phase thematic analysis framework (Braun and Clarke, 2006) was utilized. The initial data analysis began during the transcription of interviews, as comments and codes were recorded while transcribing and reviewing observation notes. All data sheets were systematically structured and organized by transferring them into the qualitative analysis tool, MAXQDA Analytics Pro 2024. Relevant data were systematically coded into comprehensible codes using the MAXQDA software. Thematic analysis, as outlined by Braun and Clarke (2006, 2019), does not require large sample sizes to generate meaningful insights. Instead, the focus is on identifying patterns and themes in the data, which can often be achieved with smaller, well-defined samples. We coded raw data examples representing a specific meaning unit (sentences, phrases, single words) and classified codes according to potential themes. Subsequently, the validity of the themes was confirmed through a meticulous examination of all codes and the entire dataset. Themes were refined and named, resulting in a definitive thematic hierarchy. The final report was written with the help of a literature review (Braun and Clarke, 2019).

The coding was performed by two researchers: the principal investigator (EA) coded all transcripts, while a researcher with expertise in data processing (EOK) independently co-coded them. When inconsistencies or conflicts in coding arose, the coders engaged in discussion until a consensus was reached. If disagreements persisted, a third referee (YB) with expertise in health and communication fields was consulted to make the final decision. The inter-coder agreement rate is 90%. To ensure reliability in the analytical process, EA, EOK, and YB held regular meetings to discuss and defend the expression and content of the codes, as well as the conceptual relationships and arrangement between codes, themes, and sub-themes. Peer debriefing was employed to enhance the reliability of the analysis. As Mason (2010) and Vasileiou et al. (2018) emphasize, smaller sample sizes are often sufficient in qualitative research as long as data saturation is achieved. The focus on depth over breadth ensures that smaller sample sizes, such as the 13 participants in our study, can still provide significant insights into the research question. During the analysis process, the researchers of this study and members of the research group engaged in discussions on codes and themes for validation of the findings. Themes were established based on an extensive review of the existing literature and a combination of codes and interpretations emerging from the analysis of previous data.

### 2.5 Ethics statement

The study has received approval from the Karabük University Social and Humanities Scientific Research and Publication Ehics Board (Decision no. 2024/02 dated January 26, 2024). All interviewed individuals were informed about the purpose and methods of the study. Written informed consent was obtained from the interviewees before starting the interviews. The files containing the interview recordings and transcripts have been coded as P1, P2, etc., to ensure confidentiality. The recordings and transcripts do not contain any personal data that could identify the individuals interviewed.

## 3 Results

The initial participants were selected using a convenience sampling method, and as the study progressed, the snowball technique was employed. The first participant was recruited through a psychologist (A.I) who had experience working with individuals who had attempted suicide, allowing us to begin the recruitment process for this sensitive topic. The interviewers conducted interviews with individuals with whom they had no close relationships, such as family or friends. As a result, a total of 13 interviews were conducted, comprising 6 women and 7 men. The age range of the participants is between 18 and 29. The participants reside in different cities. All participants had attempted suicide at least once, and these attempts were unsuccessful. Detailed sociodemographic characteristics of the study group are provided in Table 1.

**Table 1:** Detailed sociodemographic characteristics of the study group.

Participant	Age	Gender	Education	Marital status	Income rate	Occupation
Participant 1	25	Man	University	Single	Middle	Customer representative
Participant 2	22	Woman	University	Single	Middle	Journalist
Participant 3	29	Man	High school	Married	Middle	Sales assistant
Participant 4	24	Man	University	Single	Low	Student
Participant 5	25	Woman	University	Single	Middle	Instructor
Participant 6	23	Woman	University	Single	Middle	Marketing Officer
Participant 7	24	Man	University	Single	Middle	Student
Participant 8	18	Woman	High school	Single	Middle	Unemployed
Participant 9	19	Woman	High school	Single	Low	Executive Assistant
Participant 10	21	Man	University	Single	Low	Nurse
Participant 11	27	Woman	High school	Married	Low	Cashier
Participant 12	24	Man	High school	Single	Low	Unemployed
Participant 13	26	Man	High school	Single	Low	Courier

The analyses conducted concluded that data saturation was achieved, and additional interviews did not provide new themes relevant to the objectives of the study. Therefore, the number of participants was limited to 13.

**Figure 1:**
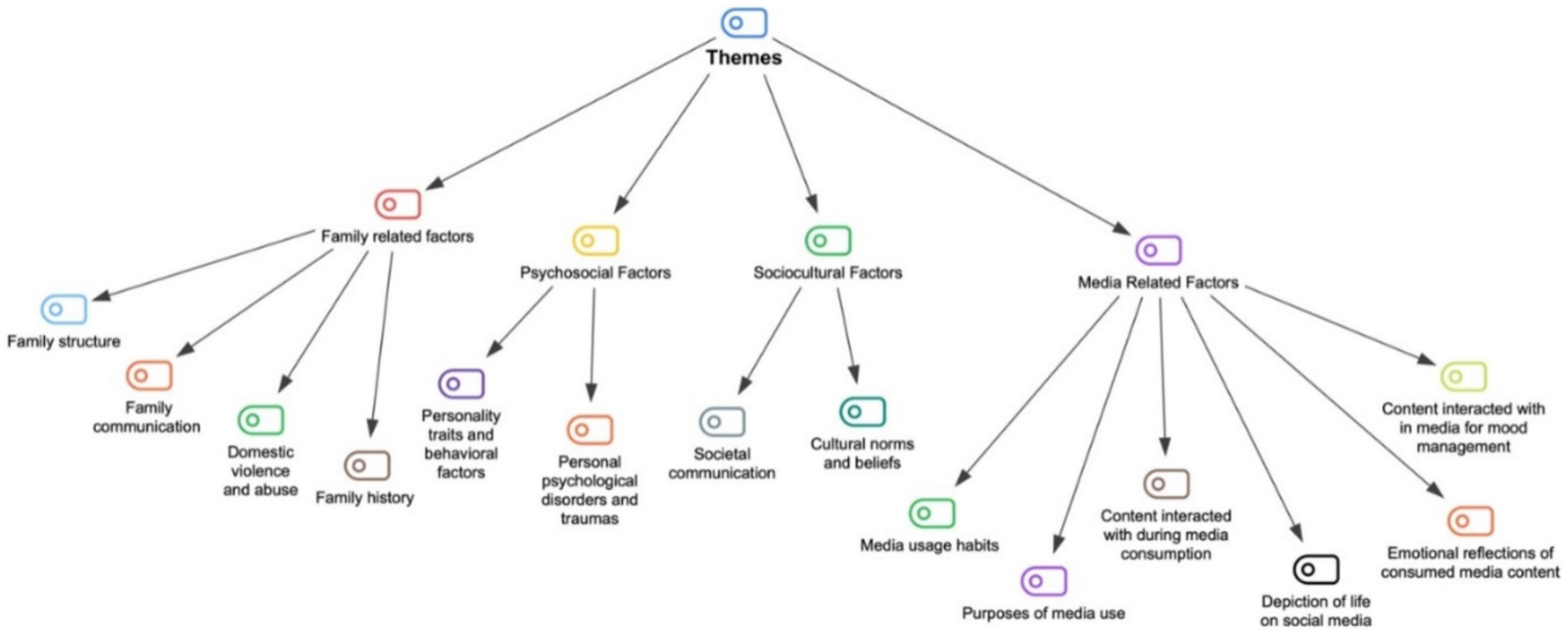
The coding tree of the thematic analysis.

The data obtained from the interviews can be summarized in four main themes: (1) Family-related factors, (2) Psychosocial factors, (3) Sociocultural factors, and (4) Media-related factors. For the first theme, four subthemes have been identified: (1) Family structure, (2) Family communication, (3) Domestic violence and abuse, (4) Family history. The second theme includes two subthemes: (1) Personality traits and behavioral factors, (2) Personal psychological disturbances and traumas. The third theme also contains two subthemes: (1) Societal communication, (2) Cultural norms and beliefs. Lastly, in the fourth theme, six subthemes have been determined: (1) Media usage habits, (2) Purpose of media use, (3) Content interacted with during media consumption, (4) Depiction of life on social media, (5) Emotional reflections of consumed media content, and (6) Content interacted with in media for mood management. All themes and subthemes are displayed in Figure 1. Each theme is explained sequentially with illustrative quotes selected from the participants’ data.

### 3.1 Family related factors

**Figure 2:**
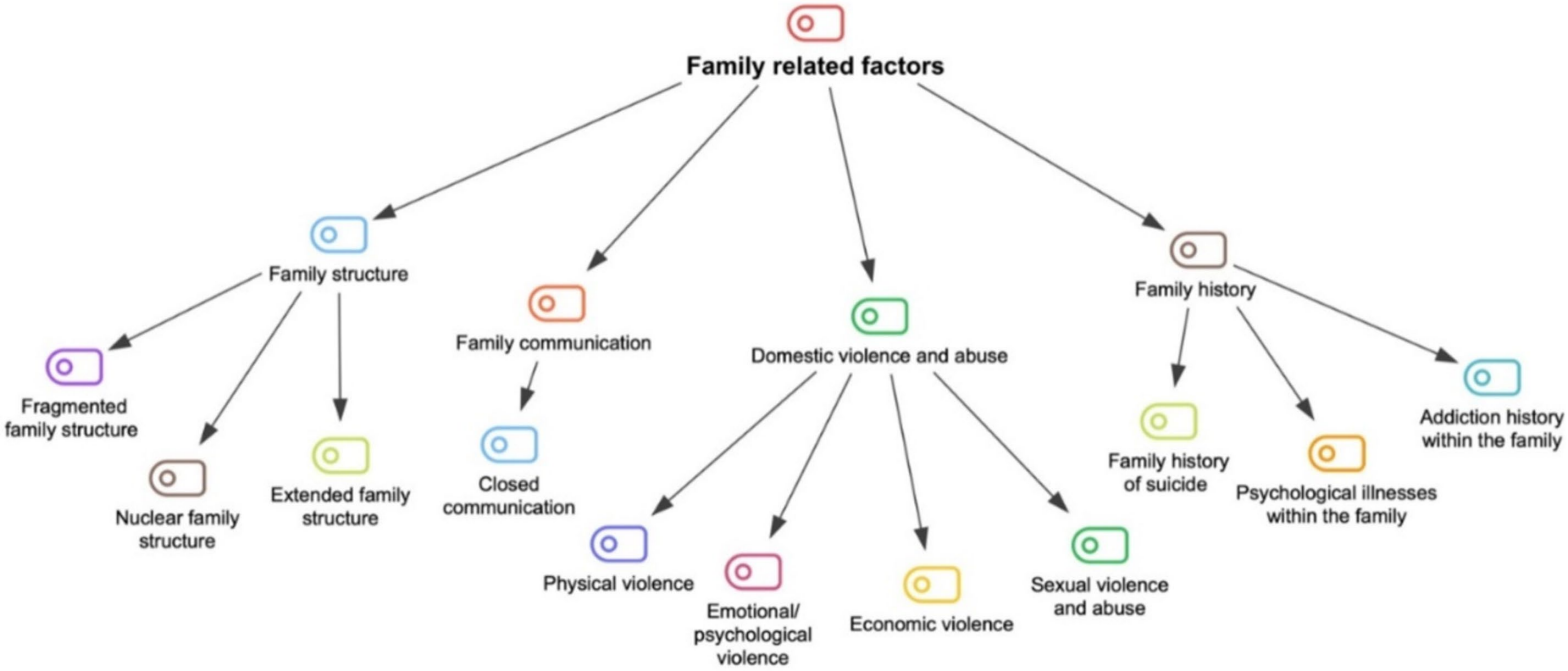
The coding tree of family related factors.

The family is the first social environment for individuals and plays a significant role throughout their lives. Family dynamics can profoundly affect individuals’ personal development, relationships, and psychological health. For this reason, four sub-themes have been identified for the first theme: (1) Family structure, (2) Family communication, (3) Domestic violence and abuse, and (4) Family history. The sub-themes of the family-related factors theme and the categories related to these sub-themes are displayed in Figure 2.

#### 3.1.1 Family structure

Family structure is a crucial factor that determines the interaction and communication style among family members. This sub-theme has been analyzed in three categories: (1) fragmented family structure, (2) nuclear family structure, and (3) extended family structure. Interviews reveal findings related to participants’ family structures, showing that many belong to fragmented families where marital or cohabitation relationships have ended:

*(P5; woman): My parents are officially married but have been living separately as long as I can remember. So, on paper, they are married. My siblings and I live with our mother.*

*(P10; man): My parents are separated. I have not seen my father for 7 years. He was abroad before that. So, I can say I hardly know him.*

*(P12; man): My parents have been separated for years. (..) I do not see my father much.*

Participants with nuclear families highlight violence and issues within the family:

*(P3; man): My parents are alive and together. However, I had a very bad childhood.*

*(P6; women): My parents are together. However, there are problems in our family, my father is oppressive.*

*(P13; man): My parents live together. (...) We had a lot of arguments at home because we had very different views on the same subject.*

One participant comes from an extended family structure. The participant living with grandparents emphasizes that this situation limits individual freedom and privacy and increases pressure:

*(P4; man): My parents are alive and we live together. We are 8 siblings. (...) We have a traditional culture. Our family structure is dominated by a clan system. So grandparents, uncles, cousins all live together. There is no privacy, you cannot move freely. It’s a family structure where everyone interferes, treats their own child like an object, devalues them. Individually, we are deprived.*

#### 3.1.2 Family communication

Family communication is extremely important for strengthening emotional bonds among family members, increasing trust, and maintaining healthy relationships. All participants describe their family communications as closed and emphasize communication issues:

*(P4; man): I do not call him father, nor her mother. I do not think they deserve it. (...) I have no emotional ties to my family. Right now, I do not miss them. (...) I do not want to know what they are doing or experiencing.*

*(P7; man): The lack of communication and love was at its peak in my family. (...) We hardly shared anything anyway.*

*(P9; woman): I do not really talk much with my parents. I usually spend time by myself in my room.*

#### 3.1.3 Domestic violence and abuse

For this sub-theme that threatens security, health and well-being within the family, four categories were determined: (1) physical violence, (2) emotional/psychological violence, (3) economic violence, (4) sexual violence and abuse. Participants are also exposed to four types of violence within the family: Physical violence refers to one family member being physically aggressive toward another. Most of the participants are exposed to this type of violence, and they describe the violence they experience with the following words:

*(P3; man): I grew up with extreme pressure and extreme beating. I was subjected to violence by my father.*

*(P7; man): There would not be a day without fighting, noise and violence in the family.*

*(P12; man): There were problems within the family, including physical violence. There were bloody knife fights between my mother and father, and between me and my brother.*

Emotional or psychological violence is when a family member psychologically harms another by methods such as humiliating or threatening. This type of violence can seriously affect the victim’s self-esteem and emotional well-being. Most participants experience this type of violence:

*(P5; woman): So, for as long as I can remember, my father has been unfaithful, uncaring and did not fulfill his parental duties. There was no physical violence, but psychological violence was always in our lives.*

*(P6; woman): It interferes with my preferences, my clothing style, and the places I go to. We have constant arguments at home.*

*(P11; woman): It is not physical violence, but we have said a lot of bad words to each other. This affected me negatively.*

Economic violence refers to one family member controlling another financially or limiting their independence. Most of the participants who expressed economic difficulties are exposed to this type of violence and emphasize the violence they suffered as follows:

*(P5; woman): My father would meet our financial needs, but if he wanted to, he would sometimes not send money for months. He caused poverty in the midst of wealth, and left him fatherless while he was still alive.*

*(P8; woman): My father was very angry. My mother wanted to work, but she did not let me. He said stay at home and take care of your children. (…) he could not meet the needs on his own. This situation had a negative impact on us.*

When a person sexually harms or abuses another person, it can lead to very deep and traumatic consequences. This type of abuse is usually carried out through means of force, control, or manipulation and without the victim’s consent. One of the participants experienced sexual violence and abuse:

*(P4; man): I was sexually abused by my own uncle for 14 years and I could not tell anyone. It is a difficult process (…). Complete helplessness, loneliness, misunderstanding, fear. (…) You have no hope, you have no life energy, you have no enthusiasm, you are tired of everything.*

#### 3.1.4 Family history

This sub-theme is divided into three categories: (1) family history of suicide, (2) psychological illnesses within the family, and (3) addiction history within the family. A family history of suicide can be a painful experience that causes deep sorrow and trauma among family members. Generally, participants have a history of suicide in their families or close environments:

*(P1; man): There are relatives who have experienced suicide. My father, my uncle.*

*(P5; woman): My cousin hanged himself in his dormitory during his university years.*

*(P6; woman): In my surroundings, there are relatives who have committed suicide or shown suicidal tendencies. My father’s aunt hanged herself; I’ve often questioned this.*

The psychological illness of a family member can leave deep impacts among family members and significantly affect family dynamics. Some participants mention psychological illnesses in their family:

*(P3; man): My father used to break my arms and legs with iron. He was a psychopath.*

*(P8; woman): My father had insomnia issues. He would watch TV, unable to sleep at all. It’s still like that; he has problems with it. We were disturbed by this.*

*(P13; man): My mother was very anxious, and this was reflected at home. (...) I know that she used medication from time to time.*

A family history of addiction can lead to serious stress and difficulties among family members. Some participants highlight an addiction history in their family:

*(P1; man): My father drinks a lot, coming home every day and tormenting us.*

*(P2; woman): My father used to gamble, which led to a lot of domestic violence. There was hardly any family structure.*

*(P12; man): My father has calmed down a bit due to his age, but he is fond of pleasure and drinks alcohol. (…) All the fights started because of alcohol.*

### 3.2 Psychosocial factors

**Figure 3:**
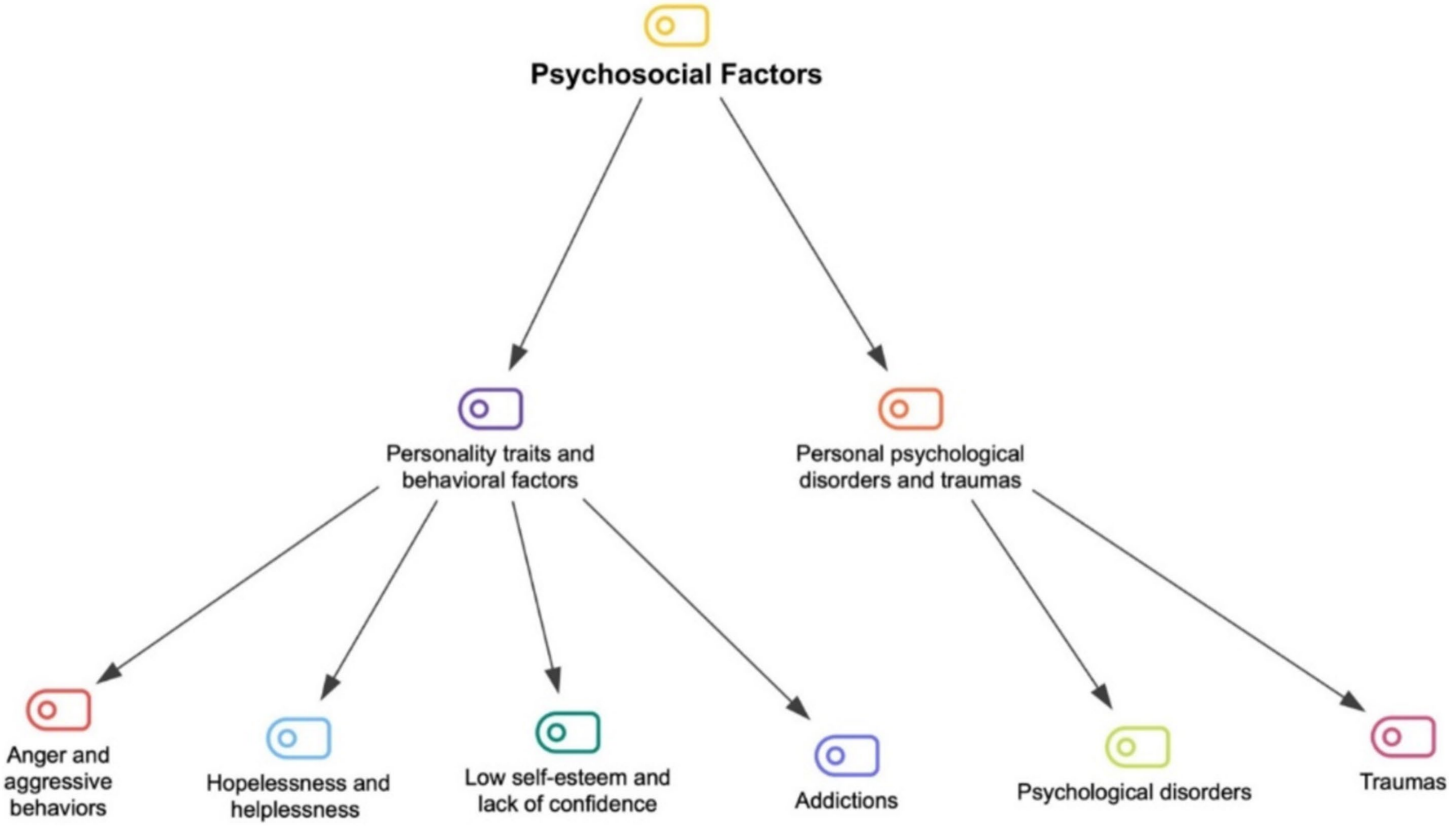
The coding tree of psychosocial factors.

Psychosocial factors are used to explain the relationship between an individual’s social environment and their psychological state. Under this main theme, there are two sub-themes: (1) personality traits and behavioral factors, and (2) personal psychological disorders and traumas. The sub-themes of the psychosocial factors theme and the categories related to these sub-themes are shown in Figure 3.

#### 3.2.1 Personality traits and behavioral factors

Personality traits and behavioral factors are significant determinants that shape how individuals think, feel, and behave. These factors profoundly affect individuals’ relationships, emotional well-being, and overall life satisfaction. Based on the data from participants, this sub-theme has been divided into four categories: (1) anger and aggressive behaviors, (2) hopelessness and helplessness, (3) low self-esteem and lack of confidence, and (4) addictions. Anger and aggressive behaviors are commonly observed symptoms among individuals at risk of suicide. Participants who express themselves often articulate this condition clearly:

*(P1; man): I try to treat myself with medications as much as I can, but sometimes (...) I have bursts of anger. (...) I cannot control myself, I lose control. Someone else comes out. I do not recognize myself.*

*(P9; woman): There is an angry and very anxious side of me. I get overly attached to people and turn it into an obsession*.

*(P12; man): My anger problems, my constantly changing and unstable moods, my constant search for flaws in myself, and my feeling of inadequacy (…) affect my life greatly.*

Hopelessness and helplessness express the emotional state where individuals generally have a negative expectation for the future and believe they cannot change their current situation. Participants indicate that hopelessness and helplessness play a central role in their actions and have led them toward suicidal tendencies:

*(P2; woman): I was at rock bottom and felt like I had reached the end of the road. I actually loved life but felt helpless. I avoided fighting. There were many moments when I thought it could not get any worse.*

*(P4; man): Maybe it’s helplessness, maybe to draw attention. I am here too. (...) See me. I wanted them to notice the pain I was living, to feel pain. I wanted to punish them. I wanted them to take responsibility.*

*(P9; woman): Due to certain events I experienced, I attempted suicide because I felt it would be better since I could not resolve the situation and felt helpless.*

Low self-esteem and lack of confidence refer to a state where an individual feels undervalued or inadequate, lacking belief in oneself. Among the participants, dissatisfaction with oneself is also a common behavioral factor. Low self-esteem and lack of confidence have been significant triggers for the suicidal act in many participants:

*(P4; man): Feelings of unlove, worthlessness make you hate yourself. Because of this, you are more prone to suicide. That’s how you start the attempt.*

*(P5; woman): I always feel my emotions very intensely. I question myself a lot and take too much responsibility in every problem, thinking it’s because of me. (...) I never like myself.*

*(P7; man): I used to value my friendships a lot and tried to make them happy. But I realized I wasn’t getting back what I gave, they did not think about me as much as I thought about them, they did not value me.*

*(P11; woman): I question myself about why I am an insecure person, but that is my habit.*

Addiction is an overwhelmingly strong desire for a substance or activity. It can affect an individual’s normal functioning and daily life, leading to personal and social problems. Most participants smoke cigarettes. Approximately half of them consume alcohol, and three of them state that they are addicted to alcohol. Five participants use marijuana/weed/drugs, and three declare themselves as addicted to smartphones/social media.

*(P1; man): I use weed and cigarettes. I also constantly use Instagram. My phone is always in my hand; it’s become a habit. (...) It never drops.*

*(P6; woman): I smoke cigarettes. I have been using them since 8th grade. I think I have a phone addiction. Several times, police have warned me about not putting down my phone when crossing the street.*

*(P10; man): I use cigarettes and alcohol but am trying to quit alcohol. (...) Besides, I used marijuana in high school and college.*

*(P13; man): I have used* var*ious substances many times before.(...) For a while I thought that I was really addicted to alcohol.*

#### 3.2.2 Personal psychological disorders and traumas

Personal psychological disorders and traumas refer to various conditions that affect an individual’s emotional and mental health. This sub-theme has been divided into two categories: (1) psychological disorders and (2) traumas. Approximately half of the participants express their opinions on this issue and state that they have psychological disorders:

*(P4; man): During the treatment process, I was diagnosed with depression, anxiety, borderline personality disorder by different doctors.*

*(P5; woman): I received psychiatric support after the suicide incident. I was diagnosed with major depression.*

*(P7; man): I discovered I had social anxiety when I started treatment. (...) I also have Agoraphobia*.

*(P11; woman): I have anxiety, I have a very anxious nature.*

Trauma refers to an event where an individual experiences significant physical or psychological danger or harm, leading to fear or distress. Such events can disrupt an individual’s normal functionality and emotional balance. Many participants’ life stories involve traumas, typically resulting from shocking and frightening experiences like violence, abuse, sudden losses, or abandonment:

*(P1; man): My life was going very well. I had everything—my job, relationship, house, car, shop, (...) one day I lost everything.*

*(P4; man): Especially the physical violence and sexual abuse from my uncle, which was constant and systematic for 14 years. That was the worst. After those events, I shut myself in my home, led an asocial life. (...) I had a very traumatic childhood. During my childhood and adolescence, I made plans to end my life.*

*(P5; woman): Unfortunately, during that period, my mother also lost her mother. She was very sad. (...) Soon after, my mother fell ill and there was a risk of cancer. This situation led to a fear of losing my mother and being utterly alone. I thought every day brought me one day closer to the day I would lose her.*

### 3.3 Sociocultural factors

**Figure 4:**
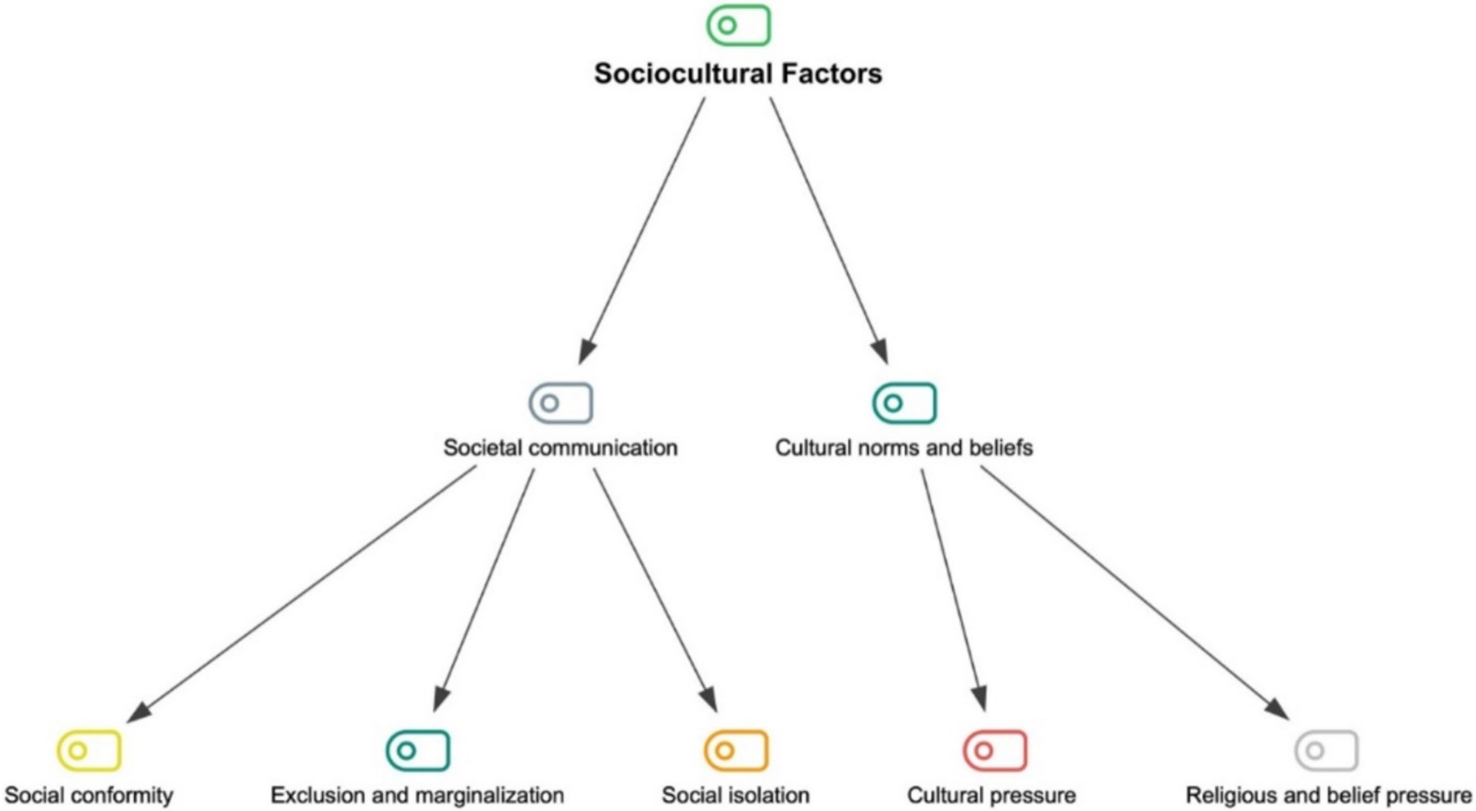
The coding tree of sociocultural factors.

Sociocultural factors refer to various elements that shape the social and cultural structures of a society and affect individuals’ behaviors, values, and relationships. They explain how individuals adapt to the values, norms, beliefs, and social structures of their society. Under this main theme, there are two sub-themes: (1) societal communication, and (2) cultural norms and beliefs. The sub-themes of the sociocultural factors theme and the categories related to these sub-themes are shown in Figure 4.

#### 3.3.1 Societal communication

Societal communication refers to the process of interaction among individuals within a society and plays a crucial role in shaping individuals’ roles, identities, and relationships. This sub-theme is divided into three categories: (1) social conformity, (2) exclusion and marginalization, and (3) social isolation.

Social conformity is the process by which individuals in a society adapt to accepted norms, values, and behaviors, shaping their interaction with the community. Many participants experience difficulties in socially conforming to their community:

*(P2; woman): I do not feel like I belong to this society. I am different. (...) My lifestyle, thoughts, and way of living are different.*

*(P6; woman): I see myself as different. My political views and my dress code do not align with my surroundings. I am against the current system. (...) I make it known to my surroundings that I keep a distance from society and have many objections. I am not in complete harmony with society.*

*(P10; man): I do not really get along with society. I often find myself holding opposing views. What they think is right is usually what I think is wrong. I get tired of trying to conform to them.*

*(P11; woman): I do not feel that I belong to this society. I think everyone lives for their own benefit.*

Participants who struggle to adapt to their society and others also face exclusion and marginalization:

*(P1; man): Even our neighbors would frown upon the tattoo on my arm. That’s why I felt excluded.*

*(P4; man): I feel excluded, looked down upon. Because I am different emotionally, intellectually, physically, in terms of clothing, religious beliefs, political views. I do not think the same as the society I live in.*

*(P7; man): I used to have long hair. I’m ginger, I have freckles, and I’m very thin. Because of my different appearance, people inevitably insult me. When insults happened, I hated society and felt marginalized.*

Social isolation refers to the tendency of an individual to avoid social interactions or weaken their social ties. Three participants have adopted a limited relationship with the people around them and an isolated lifestyle:

*(P5; woman): I have never liked the society I live in. I think they are intolerant, selfish, oppressive, gossipy, jealous. It feels like no one wishes well for others. I do not know if it’s about not fully belonging to society, maybe I prefer to stay away.*

*(P8; woman): For example, there are times in my life when I cannot even go outside, I cannot step out of the house.*

*(P13; man): In general, I do not feel like I belong to this society, people are very stupid and thoughtless. They have fixed ideas, (…) that’s why I do not establish social relations (…) I do not have a circle of friends.*

#### 3.3.2 Cultural norms and beliefs

Cultural norms and beliefs are fundamental elements that determine a society’s values, beliefs, behaviors, and social interactions. They shape the identity and social fabric of the community. This sub-theme has been divided into two categories: (1) cultural pressure and (2) religious and belief pressure.

Cultural pressure refers to the tendency of a society to impose its specific cultural norms, values, or practices on other individuals or groups. Many participants experience cultural pressure within family and societal relationships, and they find this distressing:

*(P2; woman): I feel pressured about my lifestyle from people around me (mostly from those I do not know well). This makes me very uncomfortable.*

*(P9; woman): I constantly feel under pressure. I am always concerned about what people will think about my behavior or the way I dress, whether they will talk about me, or judge me.*

*(P10; man): At home, my mother interferes with my lifestyle and clothing. (...) At work, I also feel cultural pressure. I see myself as different from the general society.*

Religious and belief pressure involves the tendency of an individual or a group to force another individual or group to adhere to a specific religion or belief system or to apply pressure against these beliefs. Three participants have been intensely subjected to religious and belief pressure, emphasizing the impact of this imposition on themselves:

*(P4; man): My family/clan is extremely oppressive, ultra-conservative. (...) They are also very religious. They pray when they lie down and get up. They tell us to pray when we lie down and get up. Until a certain period, I performed my religious duties under family pressure, but now I do not.*

*(P7; man): My father’s family comes from people important in religious circles. (...) They have been devout for generations. They live their religion and perform their religious duties. They want us to do the same. There is an inevitable pressure. (...) They impose their religion and beliefs on us. Due to my family, I have come to hate religion. I am an agnostic*.

*(P11; woman): Even though my family is very conservative, I cannot be like them. I always feel this pressure, but I do not feel like it.*

### 3.4 Media related factors

**Figure 5:**
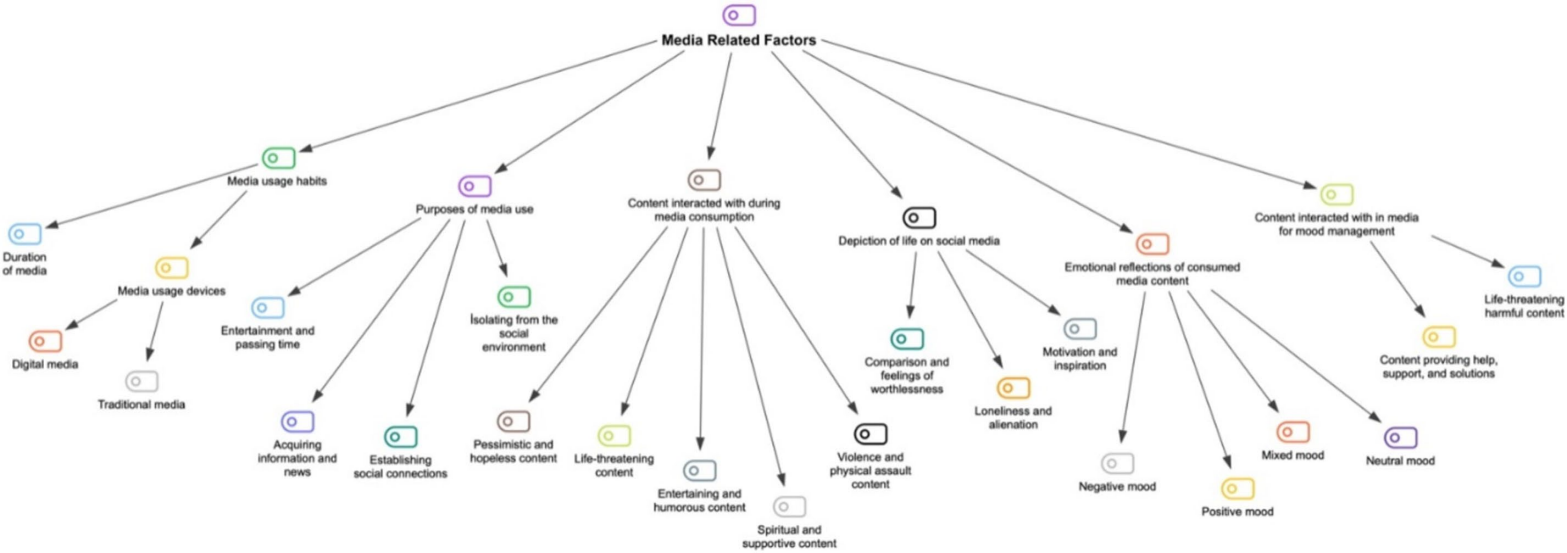
The coding tree of media related factors.

Media has the power to shape the thoughts, behaviors, and perceptions of individuals and societies and can significantly influence the general opinions and behaviors of a community. However, the impact of media is complex and varies among individuals. Factors such as personal experiences, values, beliefs, and education shape how individuals respond to media messages. Under this main theme, six sub-themes have been identified: (1) media usage habits, (2) purposes of media use, (3) content interacted with during media consumption, (4) depiction of life on social media, (5) emotional reflections of consumed media content, and (6) content interacted with in media for mood management. The sub-themes of media-related factors and the categories related to these sub-themes are shown in Figure 5.

#### 3.4.1 Media usage habits

Media usage habits have been categorized into (1) duration of media use and (2) media usage devices. According to data obtained from interviews, most participants heavily use media, consuming media content for an average of 6–7 h per day. The longest media usage among participants is 16 h, and the shortest is 1 h. All participants who use media heavily prefer digital media as their media usage device. Participants who do not have a regular practice of using traditional media read books as a traditional media device. Media usage habits are listed in Table 2.

**Table 2:** Media usage habits.

Media usage habits			
Participants	Duration of media use	Media usage devices	
		Digital media	Traditional media
P1	10 h/per day	Facebook, Instagram, Spotify, X, vkontakte, WhatsApp, Youtube	Book
P2	5 h/per day	Instagram, X, WhatsApp, Youtube	Book
P3	4–5 h/per day	Facebook, Instagram	–
P4	16 h/per day	Facebook, Instagram, Spotify, X, WhatsApp, Youtube, Tinder, Telegram	Book
P5	7–8 h/per day	Instagram, Spotify, X, WhatsApp, Youtube	Book
P6	8–9 h/per day	Instagram, TikTok, WhatsApp	Book
P7	2–3 h/per day	Facebook, Instagram, Spotify, X, Xbox, WhatsApp, Youtube	Book
P8	1 h/per day	Instagram, WhatsApp	–
P9	10 h/per day	Instagram, TikTok, X, WhatsApp	–
P10	4–5 h/per day	Instagram, Spotify, Telegram, X, WhatsApp	Book
P11	5–6 h/per day	Facebook, Instagram, Spotify, X, WhatsApp, Youtube	–
P12	8 h/per day	Facebook, Instagram, TikTok, Spotify, X, WhatsApp, Youtube	–
P13	4–5 h/per day	Instagram, TikTok, X, WhatsApp, Youtube	–

#### 3.4.2 Purposes of media use

The purposes of media use among participants have been shaped by interviews and categorized into four headings: (1) entertainment and passing time, (2) acquiring information and news, (3) establishing social connections, and (4) isolating from the social environment. Most participants describe their purpose for using media as entertainment and passing time:

*(P1; man): My purpose on social media is to follow popular topics, popular people, and to fill the void, basically to pass time.*

*(P2; woman): I use it to pass the time and to see what’s going on, but I get bored after a while.*

*(P3; man): I look at it to distract myself from my troubles, to be occupied with something else.*

*(P12; man): I use social media to temporarily forget the psychological problems in my head, which are caused by emptiness.*

One participant expresses the purpose of media use as acquiring information and news:

*(P10; man): I generally follow pages related to scientific research. I use Instagram for this. I follow health pages and pages that describe what to do in emergencies. (…) This way, instead of mindlessly scrolling through social media, it adds something to me.*

Two participants mention that, in addition to entertainment and passing time and acquiring information and news, they use media for establishing social connections:

*(P5; woman): On X, I follow the current affairs. On Instagram, I follow content related to celebrities, travel,* etc. *I use YouTube for movies and series. I intensely watch people’s lives on social media platforms. I’m curious about who is doing what, with whom, and where. I also engage with them.*

*(P9; woman): I follow informative and political content on social media. (...) Through apps like TikTok, Instagram,* etc.*, I frequently spend my active free time to clear my mind. Mostly, I look at what people are doing on social media, follow their posts.*

Two participants prefers to use media to isolate from the social environment; that is, they deliberately use media to distance themselves from the outside world and environmental influences:

*(P7; man): I prefer not to engage in social relationships in everyday life; I struggle a lot with relationships. I do not use social media to socialize. On the contrary, I use it to isolate myself from the environment. I do not even follow the news. I want to stay away from current affairs. I only watch reels, cat and dog videos.*

*(P13; man): Since I do not have a circle of friends, I like spending time on social media more. I would rather stay at home and surf the internet than listen to their empty conversations. (…) I am aware that I isolate myself from society. But I’m happier this way.*

#### 3.4.3 Content interacted with during media consumption

The content with which participants engage during media consumption is classified according to their choices in movies, series, music, books, blogs, and social media preferences. Based on their responses, five categories have emerged: (1) pessimistic and hopeless content, (2) life-threatening content, (3) entertaining and humorous content, (4) spiritual and supportive content, and (5) violence and physical assault content.

Although most participants cite entertainment and passing time as their primary media use, the content they interact with prominently features pessimistic and hopeless content, and life-threatening content:

*(P1; man): When I feel bad, I watch videos, which are generally of a pessimistic nature.*

*(P4; man): I often come across depressive, gloomy quotes, poems, suicide-related posts on my feed. There was a time when my explore page was full of such pages. I constantly read them and find myself falling into depression.*

*(P11; woman): I watch family dramas, loneliness, stories of people who have suffered in life, and people who cannot make their voices heard. (…) I do not feel like watching hopeful, exciting, joyful things.*

Life-threatening content includes physical, mental, or emotional health-threatening materials, covering elements such as violence, harassment, suicide, promotion of dangerous habits, and hate speech. Some content, particularly harmful to the youth, can severely impact mental health and even contribute to suicide risk. Two participants have explicitly described the content they interact with as life-threatening:

*(P4; man): There’s a series called ‘13 Reasons Why.’ (...) It discusses peer bullying, suicide, sexual abuse. A girl commits suicide. After her death, she leaves behind 13 tapes. Each tape describes people who left her alone, harmed her. That series plunged me back into depression because every episode describes what I’ve gone through. (...) I thought, I could do what she did in the series.*

*(P5; woman): During the period I attempted suicide, I extensively followed content related to suicide on YouTube, Instagram, and X, even getting lost in it.*

Four participants engage with entertaining and humorous content in their media consumption, attempting to distract from their issues:

*(P2; woman): Initially, I was very troubled and interested in violent content videos. But now they no longer interest me. Negative content bothers me, so I’ve been choosing comedy series and entertaining content lately.*

*(P7; man): I cannot watch series that contain violence, suicide content; they stress me out too much. (...) I do not even follow the news anymore; to avoid negative content, I prefer funny, lighthearted content.*

*(P13; man): I love speed. That’s why I mostly watch motorsport-related activities. (…) I generally engage in funny and entertaining content.*

Spiritual and supportive content includes materials that enhance or strengthen an individual’s mental well-being, inner peace, and motivation. Such content is created with the aim of spiritual healing, positive thinking, and improving quality of life. Two participants mention interacting with spiritual and supportive content:

*(P3; man): On social media, I often come across beneficial sayings. They spiritually affect me, and I feel relaxed when I read them.*

*(P5; woman): I read books related to my field. I try to read personal development books but never manage to apply them.*

Two participants interact with content primarily featuring physical, verbal assaults, harassment, beatings, and bullying:

*(P6; woman): On TikTok, suicide, cutting, stabbing scenes often appear. I had liked some videos before. Then they started appearing more often. (...) Suicide and injury scenes from movies come up. These scenes negatively affect me, make me feel worse, and lower my spirits.*

*(P9; woman): Sometimes violent content videos pop up, and I’ve watched them a few times. They still appear. I do not want to watch too much, but it feels like there’s nothing else.*

#### 3.4.4 Depiction of life on social media

Depiction of life on social media refer to how individuals represent themselves and their lives on online platforms. This includes how they portray themselves, what aspects they highlight, and what they choose to conceal. Often, life depictions on social media can include idealized, filtered, or selectively curated versions that may not fully reflect real life. All participants describe their media usage habits as digital, with the majority spending long hours on social media. The interviews have resulted in three categories under this sub-theme: (1) comparison and feelings of worthlessness, (2) loneliness and alienation, and (3) motivation and inspiration. Nearly all participants compare themselves to the life depictions on social media and feel worthless:

*(P5; woman): The lives I see on social media negatively affect me. I feel like I’m both aspiring to be like them and facing my deficiencies, constantly feeling inadequate. It’s as if everyone has everything, and I have nothing. (...) I feel a lack of desire to live and hopelessness.*

*(P6; woman): Seeing the luxurious life on social media, not having to work and being able to buy anything, go anywhere, it depresses me. (...) They do not have financial issues. Despite working, I cannot afford the things I want or go places I wish to. I question myself why.*

*(P11; woman): Everyone is very rich on social media, everyone is very friendly, everyone is very peaceful. I look at myself and I am not this peaceful. (…) I do not find myself very valuable.*

Those who compare themselves and feel worthless due to social media life depictions also tend to experience feelings of loneliness and alienation:

*(P1; man): Sometimes when I feel lonely, I go on social media. Other times, while on social media, I realize how alone I am, especially when I see my friends together.*

*(P4; man): Considering I consume media for 16 h a day, of course, it increases my feeling of loneliness. Just me and my phone, how much more alone can one be?*

*(P12; man): I feel worthless and lonely. I keep thinking that the life I’m living is not worth living.*

However, two participants mention that depictions of life on social media have a positive impact on them, serving as encouragement. The content they see on platforms inspires them to make positive changes in their lives and provides motivation and inspiration:

*(P3; man): The content on social media, at least what I follow, distracts me from overthinking and negative thoughts. The pages I follow are somewhat spiritual. (...) They say things like “Hang in there, beautiful days are waiting for you. Bright days are ahead.” (...) I keep finding life-giving words one after another.*

*(P8; woman): I take inspiration from Britney Spears; her experiences had a big impact on me. (...) They tried to put her in a mental hospital. I had gone through similar things. Even my mother had thought about “whether we should hospitalize her.” Seeing how she got through her situation, found something to hold onto affected me (...) it made me happy. At that moment, despite my problems, I felt like I could move forward somehow.*

#### 3.4.5 Emotional reflections of consumed media content

Mood refers to the emotional state experienced by an individual at a particular time, typically lasting a few hours to several days. The emotional responses individuals experience while consuming media content are often dependent on how that content interacts with their personal experiences, interests, and emotional state. This sub-theme examines the reflections of consumed media content on participants’ moods and has been divided into four categories: (1) negative mood, (2) positive mood, (3) mixed mood, and (4) neutral mood. Most participants experience a negative mood in response to the media content they consume:

*(P5; woman): Content shared by people about the meaninglessness of life seemed to describe me. During that period, social media had the most negative impact on me; I could not look up. It affected my entire daily routine.*

*(P10; man): The ideal lives people present on social media actually make me question myself. The lifestyle presented as ideal on social media does not align with my life and ideas.*

*(P11; woman): The ideal life shown there affects me negatively. Because I do not see myself at that level. Everyone talks about psychological health. I do not fit any of them.*

One participant experiences a positive mood in response to the media content consumed:

*(P8; woman): Since I do not consume much media, I cannot say it negatively affects me. When I researched Britney Spears’ life story in the media, her achievements gave me hope.*

Some participants experience a mixed mood in response to the media content they consume:

*(P3; man): It actually helps me. Yes, social media helps me get rid of negative thoughts. I do not think about anything. (…) Sometimes I let myself go, meaning I feel sad. I enter a mode of ‘whatever happens, happens, enough is enough’.*

*(P6; woman): I listen to rap music, especially tracks with swearing and insults, to let off steam. I listen to them against people I’m angry with. Sometimes, I also (…) watch comedy movies, series, and videos to distract myself from my own mood.*

*(P12; man): When I see people living luxurious lives, I often become pessimistic and depressed. (…) But when I watch motivational videos about sports, on the contrary, I feel at peace and my morale increases.*

Three participants experience a neutral mood in response to the media content they consume:

*(P1; man): I used to look at the beautiful lives shared on social media and wish I could be there, do what they do. It seemed nice. Now I realize those lives are fake, so they no longer affect me.*

*(P7; man): I’m not very affected by external factors; the music I listen to, the books I read, or the movies and series I watch do not really influence my emotions.*

*(P13; man): What I see on social media does not affect me. (…) There is no change in my emotional state, I laugh at what I see, I am not affected.*

#### 3.4.6 Content interacted with in media for mood management

Mood management refers to an individual’s ability to identify, understand, and regulate their emotional responses. It focuses on the capability to reduce negative emotions or enhance positive ones. When used correctly, media can provide emotional support or improve emotional states, but it can also negatively impact emotional states if used improperly. Under this sub-theme, two categories have emerged: (1) content providing help, support, and solutions, and (2) life-threatening harmful content.

Only three participants turn to content that provides help, support, and solutions while engaging with media for mood management:

*(P1; man): Whenever I feel down, I listen to music on Spotify. For instance, I believe music calms me down a bit. It could be slow tracks or sometimes energetic, Turkish rap songs when I’m very cheerful.*

*(P3; man): When I’m in a negative emotional state, I go on social media looking for solutions, and what I find are words that keep me alive. I do not do any other research.*

*(P12; man): Content containing help, support and solutions are more prominent. (…) I listen to a lot of music, one of the two or three things I enjoy in life and the most important one is music. The dreams I have about this field connect me to life.*

Most participants, however, engage with life-threatening harmful content for mood management:

*(P4; man): I did not seek help, but being prone to suicide, and using media frequently, I searched on Google for ways to commit suicide. How can I commit suicide? (...) I asked artificial intelligence. How can a person kill themselves? (...) I researched a lot about which method can kill faster and easier. I looked at blogs written about it.*

*(P5; woman): When negative thoughts come to mind, I did not search the internet for solutions to cope with my feelings. Rather, I used the internet or social media not to find a cure for my depression, but to intensify my confusion or materialize my suicidal thoughts during depressive times.*

*(P6; woman): I also thought about jumping off a building. (...) I researched it in the media. Jumping height, the way to jump, etc… For instance, head-first (head-down) jumping is more certain, landing on feet reduces the chance of dying. (...) I researched train suicides, watched videos about jumping in front of a train.*

## 4 Discussion

The purpose of the study is to investigate how communication processes and media consumption habits may influence suicidal thoughts among young adult males and females, aged 18–29, who had attempted suicide at least once. The data obtained from the interviews have been summarized in four main themes: (1) Family related factors, (2) psychosocial factors, (3) sociocultural factors, and (4) media related factors. For the first theme, four subthemes were identified: (1) Family structure, (2) family communication, (3) domestic violence and abuse, (4) family history. The second theme includes two subthemes: (1) personality traits and behavioral factors, (2) personal psychological disorders and traumas. In the third theme, there are two subthemes: (1) Social communication, (2) cultural norms and beliefs. Finally, the fourth theme is divided into six subthemes: (1) media usage habits, (2) purposes of media use, (3) content interacted with during media consumption, (4) depiction of life on social media, (5) emotional reflections of consumed media content, and (6) content interacted with in media for mood management. The data from the interviews have been summarized under these four main themes, relevant subthemes, and explanatory categories.

Family-related factors constitute the first main theme of the study. The subtheme of family structure, a crucial factor determining interactions among family members, has been examined as fragmented, nuclear, and extended family structures. In response to RQ1, the study found that family structure and communication patterns within the family were significant contributors to suicidal tendencies. Most participants came from fragmented families, with high levels of conflict and violence, which exacerbated their emotional distress. Research on suicide shows that family structure is extremely decisive in influencing individuals’ inclinations toward suicide (McLaughlin et al., 2014; Brent and Melhem, 2008; Brent and Mann, 2005; Wagner et al., 2003; Turecki et al., 2019). In this research, most participants come from fragmented family structures. Some non-fragmented families also reported facing issues of violence and conflict, which contributed to suicidal tendencies in individuals. One participant, living within a traditional culture’s tribal system, has described their family structure as lacking privacy, limiting freedom, and excessively intrusive, framing it as lacking individuality and highlighting the enduring problems this creates. These findings align with the literature, which suggests that poor family communication and structure can increase the likelihood of suicidal behaviors.

In addition to family structure, the way families communicate also plays a crucial role. Nearly all participants reported poor communication within their families, describing their family environments as closed and emotionally distant. This finding aligns with previous studies indicating that communication breakdowns within families are a significant factor in suicidal behavior (Lindström and Rosvall, 2015; Trovato, 1987; Lee, 2022; Creuzé et al., 2022). Thus, the study supports RQ1 by demonstrating how both the structural characteristics of families and their communication dynamics contribute to an increased risk of suicide.

Participants in the subtheme of domestic violence and abuse have reported experiencing physical, emotional, economic, and sexual violence within the family. Literature indicates that domestic violence and abuse increase the risk of suicide (Afifi et al., 2009; Brent and Mann, 2005; Molnar et al., 2001; Durkheim, 2013). Findings also address RQ1 and demonstrate how these negative family interactions, combined with poor communication, create an environment that increases the risk of suicidal ideation and behavior.

Participants have shared experiences of physical violence and pointed to its negative effects. They have also reported psychological violence, emphasizing its detrimental impact on their and their family’s emotional well-being. Economic insufficiencies are recognized as a significant cause of suicide in international literature (Alvarez-Galvez et al., 2017; Rivera et al., 2017; Tolga et al., 2023; Hu et al., 2020; Marcotte and Hansen, 2024). Some participants have mentioned experiencing economic hardships, stating that this situation has made them unhappy. There is a participant who has experienced systematic sexual violence, leaving profound effects on their life; however, this type of violence has not been mentioned by other participants.

In the subtheme of family history, family suicide history, psychological disorders, and addictions have been focused on. Studies in the literature show that suicides within the family or close circle can have encouraging effects and may increase suicidal tendencies (Agerbo et al., 2002; McLaughlin et al., 2014; Brent and Melhem, 2008; Hawton et al., 2002). It has been observed among participants that suicide cases are frequent within their families or surroundings, and there are psychological disorders and issues like alcohol dependency within the family members.

The interaction between individuals’ social environment and their psychological state is addressed under the second main theme of the study, psychosocial factors. In the study conducted for RQ2, it was found that personality traits such as aggression, hopelessness and low self-esteem were frequently observed in suicidal individuals. The subtheme of personality traits and behavioral factors includes anger and aggressive behavior, hopelessness and despair, low self-esteem and lack of confidence, and addictions, where extensive literature has been found (Fromm, 2017; Schopenhauer, 2013; Durkheim, 2013). Various studies show that anger and aggressive behaviors can increase the tendency toward suicide (Hill et al., 2020; Dillon et al., 2020). Additionally, factors such as hopelessness, despair, and lack of confidence are emphasized in academic literature as increasing individuals’ propensity for suicide (Brott and Veilleux, 2024; Liu et al., 2020; Grigienė et al., 2022). Aggressive behaviors are commonly observed among participants. They often experience feelings of anger and struggle to control this emotion continuously. Hopelessness and despair are identified as commonly seen personality traits. Participants have expressed feeling helpless when unable to make desired changes, and this feeling leads to suicidal thoughts. Lack of confidence and dissatisfaction with themselves are also common among participants, while feelings of unloved and worthlessness are frequently expressed. Moreover, most participants possess various addictions that could affect their daily lives and social relationships. Smoking is common among participants; about half of them consume alcohol, with three of them stating they are alcohol-dependent. Five participants use marijuana or other drugs, and three describe themselves as addicted to smartphones or social media. These addictions contribute to worsening life quality and increasing suicidal tendencies (Carballo et al., 2006; Wang et al., 2023; Conner et al., 2014; Giesbrecht et al., 2024; Yeskendir et al., 2023).

In another subtheme, personal psychological disorders and traumas affecting an individual’s emotional and mental health were examined. According to international literature, traumas and severe events can lead individuals to suicide (Fergusson et al., 2000; Rubenstein et al., 1989; Caro-Cañizares et al., 2024; Armoon et al., 2024). Half of the participants realized that they had psychological disorders such as depression, anxiety, borderline, and social anxiety during their treatment process after a suicide attempt. Moreover, many of the participants’ life histories include traumatic experiences, typically resulting from violence, abuse, sudden losses, or abandonment. One participant expressed having a highly traumatic childhood due to abuse from an early age and clearly stated that during this period, they harbored thoughts of ending their life. This finding directly answers RQ2, as it can be said that personality traits and psychological disorders have an important role in influencing suicidal behavior.

Regarding RQ3, the study found that societal pressures, cultural norms, and feelings of social isolation played a significant role in participants’ suicidal thoughts.

Sociocultural factors, which shape the social and cultural structures of a society and influence individuals’ behaviors, are the third main theme of the study. The subtheme of social communication has been considered in terms of social adaptation, exclusion, and alienation, and social isolation. Participants frequently felt alienated due to societal expectations, particularly around family and religious norms. Numerous studies exist suggesting that social isolation and social maladaptation can trigger suicide (Stellino, 2020; Adler, 2024; Wiglesworth et al., 2022; Durkheim, 2013). Most participants state that they have difficulty in adapting to society. These difficulties can lead to experiences such as exclusion and alienation. Some participants adopt an isolated lifestyle by forming limited relationships with their surroundings, attempting to balance this situation. These findings are consistent with the literature, which indicates that cultural pressures and social isolation increase the risk of suicidal behavior. The second subtheme under sociocultural factors, cultural norms and beliefs, has been examined in the context of cultural pressure and religious and belief pressure. Most participants are subjected to cultural pressure in family and social relationships, feeling uncomfortable and experiencing cultural division. Some participants consistently and intensely feel the pressure of religion and beliefs. The literature often discusses the relationship between religion and suicide, typically finding that religion acts as a deterrent factor preventing self-destruction (Zirojević and Marković, 2020). However, some participants feeling intense religious pressure report that this pressure causes them to reject religious values, develop hatred, and create a profound perspective difference. This addresses RQ3, as it shows that societal communication patterns and the weight of cultural norms can exacerbate suicidal tendencies, particularly when individuals feel disconnected from or pressured by their social environments.

As for RQ4, the study found that participants’ media consumption, particularly exposure to pessimistic and life-threatening content, exacerbated their suicidal thoughts.

Media related factors constitute the fourth main theme of the study. The subtheme of media usage habits has been examined and categorized into media usage duration and media usage tools. Participants consume media content for an average of 6–7 h a day, with all of them preferring digital media as their tool of media usage. Their traditional media preferences generally lean toward reading books. The subtheme of media usage purpose has been shaped into entertainment and passing time, information gathering and news consumption, forming social connections, and isolating from the social environment. Most participants have stated that their purpose for using media is for entertainment and passing time. The third subtheme, content interacted with during media consumption, has been analyzed based on preferences for films, series, music, books, blogs, and social media. Categories have emerged as pessimistic and hopeless content, life-threatening content, entertaining and humorous content, spiritual and supportive content, and violence and physical assault content. Although most participants state they use media for entertainment and passing time, the most interacted content in their media consumption has been identified as pessimistic and hopeless content and life-threatening content. Research on individuals prone to suicide has shown that pessimistic content can lead individuals to suicide (Czeisler, 2020; Pirkis et al., 2018; Niederkrotenthaler et al., 2020; Park et al., 2012).

The depiction of life on social media expresses how individuals represent themselves on online platforms. This includes highlighted or hidden aspects and can be manipulative. Often, the depiction of life on social media may not fully reflect real life. However, numerous studies indicate that messages emanating from the media universe can have a negative impact on individuals’ psychologies (Schmidtke and Schaller, 2000; Balt et al., 2023). All participants describe their media usage habits through digital media, and the majority spend long hours on social media. The subtheme depiction of life on social media has been examined within the categories of comparison and feeling worthless, loneliness and alienation, and motivation and inspiration. Nearly all participants compare themselves to the life portrayals on social media and feel worthless. Almost all of them also experience feelings of loneliness and alienation. However, two participants mention that the life depiction on social media have had a positive impact on them and have been motivational. The content they see on platforms inspires them to make positive changes in their lives and provides motivation and inspiration to achieve better. These findings answer RQ4 by demonstrating the dual role that media can play in either exacerbating or alleviating suicidal tendencies, depending on the type of content consumed and how it interacts with the individual’s emotional state.

Mood expresses an individual’s emotional state at a particular moment and typically describes an emotional condition that lasts for several hours or days, reflecting the person’s overall emotional status. The emotional responses individuals exhibit while consuming media content are generally dependent on how that content interacts with their personal experiences, interests, and emotional states. This subtheme, examining the mood reflections of consumed media content, focuses on negative mood, positive mood, mixed mood, and neutral mood. Most participants experience a negative mood due to the media content they watch (Wongkoblap et al., 2017). Some participants exhibit a mixed mood, three participants experience a neutral mood, and one reports a positive mood response to the media content consumed.

Mood management refers to the ability to identify, understand, and control one’s emotional responses. This process focuses on reducing negative emotions or enhancing positive emotions. Media can provide emotional support or improve emotional conditions when used correctly, but can adversely affect emotional states if misused. In this context, the subtheme of content interacted with in media for mood management is examined in two categories: content that includes help, support, and solutions, and harmful content that threatens life. Only three participants turn to content that includes help, support, and solutions in managing their moods. Most interact with harmful content that poses life threats in mood management. Our findings emphasize that while media can contribute to negative emotional states, it also holds the potential for positive interventions, such as raising awareness and providing emotional support for at-risk individuals. Producing quality content in media to increase awareness for suicide prevention and developing strategies is of great importance. Social media has also become an effective tool for identifying beliefs and attitudes related to suicide (Keating and Rudd-Arieta, 2021; Turkish Statistical Institute, 2023; Kirchner and Niederkrotenthaler, 2024).

### 4.1 Study strengths and limitations

The study has several significant strengths. Firstly, the research examines suicidal tendencies not only from a psychological or medical perspective, but also across multiple interdisciplinary factors such as social, cultural, and media interactions. This approach provides a better understanding of the multidimensional nature of suicidal tendencies. This research not only reinforces information in the literature but also presents unique findings compared to similar studies, particularly in cultural and geographical contexts. Additionally, the fact that the number of participants is equal to men and women increases the generalizability of the study in terms of gender differences in suicide attempts. Morever, the results uniquely highlight the diversity in perceptions of the relationship between religion and suicide. While literature generally notes religion as a deterrent to suicide, this study reveals that intense religious pressure could increase suicidal tendencies through effects like rejection and hatred of religious values.

Despite its strengths, this exploratory study has some limitations. Firstly, the selection of participants was done through convenience sampling and snowball techniques, which might have restricted the diversity of the participants. Convenience sampling can introduce sampling bias, as the participants may share similar characteristics, limiting the generalizability of the findings to populations outside the sample group. Data obtained through these methods may not fully represent the general population. Additionally, snowball sampling can also lead to sampling errors, as participants are more likely to refer individuals with similar traits, which may further reduce the diversity of the sample. Also, as the study is limited to only 13 participants, the generalizability of the findings is constrained. Furthermore, the participants in this study were predominantly young adults (ages 18–29) with high levels of education (54% holding university degrees). This homogeneity in age and education limits the ability to generalize the findings to the broader Turkish population, which is more diverse in terms of age, educational attainment, and professional backgrounds. Future research should aim to include a larger and more diverse sample group to better represent various sociodemographic characteristics, such as age, education level, and professional commitment. The use of larger and randomized sampling techniques in future research would help reduce potential biases and increase the external validity of the findings. Secondly, there might be uncertainties regarding the sincerity and honesty of participants in discussions on a sensitive topic like suicide. Participants might not have shared some information fully due to social pressure or personal concerns. Thirdly, the study considered only specific sociodemographic characteristics and did not collect information about the medical histories of the participants. This could lead to overlooking other significant factors that could influence suicidal tendencies.

## 5 Conclusion

This research has thoroughly analyzed the factors influencing individuals’ suicidal tendencies and the role of media on these tendencies. Through in-depth interviews, it has identified the impacts of complex relationships among personal, familial, and socio-cultural factors on suicidal thoughts and behaviors. One of the key findings is how digital media can shape individuals’ mental health. The study demonstrates how media consumption patterns and interactions on social media platforms can affect individuals’ emotional states positively and negatively.

Another significant outcome from the research is the variability in how individuals perceive and react to media content. This highlights the importance of tailored interventions and support mechanisms for individuals vulnerable to suicidal thoughts. Additionally, by emphasizing the impact of the socio-cultural context on suicidal tendencies, the study underscores the necessity for cultural sensitivity in research and interventions.

The themes identified in this research provide a robust framework for understanding the interplay between media usage and mental health. These findings offer valuable guidance for future research, particularly on how media can be leveraged to support at-risk individuals more effectively. Moreover, this study contributes to the ongoing conversation about creating a healthier media environment, one that both mitigates the risks of harmful content and maximizes the potential for positive mental health interventions.

## Data Availability

The raw data supporting the conclusions of this article will be made available by the authors, without undue reservation.
