# Supplementary material for: Communication dynamics and media interactions of young adults who have attempted suicide: a qualitative thematic analysis
Source: Front Psychol. 2024 Nov 1;15:1460348. doi: 10.3389/fpsyg.2024.1460348 (PMC11565212; doi:10.3389/fpsyg.2024.1460348)
Supplement: Supplementary file 1 [file Table_1.DOCX]

Supplementary Material

| **The COREQ form** | | | |
| --- | --- | --- | --- |
| **Topic** | **Item No** | **Guide Questions/Description** | **Reported on page no** |
| **Domain 1**  **Research team and reflexivity** | 1. Interviewer/facilitator | Which author/s conducted the interview or focus group? |  |
|  | 1. Credentials | What were the researcher’s credentials? |  |
|  | 1. Occupation | What was their occupation at the time of the study? |  |
|  | 1. Gender | Was the researcher male or female? |  |
|  | 1. Experience and training | What experience or training did the researcher have? |  |
|  | 1. Relationship established | Was a relationship established prior to study commencement? |  |
|  | 1. Participant knowledge of the interviewer | What did the participants know about the researcher? e.g. personal goals, reasons for doing the research |  |
|  | 1. Interviewer characteristics | What characteristics were reported about the inter viewer/facilitator? e.g. Bias, assumptions, reasons and interests in the research topic |  |
| **Domain 2**  **Study design** | 1. Methodological orientation and Theory | What methodological orientation was stated to underpin the study? e.g. grounded theory, discourse analysis, ethnography, phenomenology, content analysis |  |
|  | 1. Sampling | How were participants selected? e.g. purposive, convenience, consecutive, snowball |  |
|  | 1. Method of approach | How were participants approached? e.g. face-to-face, telephone, mail, email |  |
|  | 1. Sample size | How many participants were in the study? |  |
|  | 1. Non-participation | How many people refused to participate or dropped out? Reasons? |  |
|  | 1. Setting of data collection | Where was the data collected? e.g. home, clinic, workplace |  |
|  | 1. Presence of non-participants | Was anyone else present besides the participants and researchers? |  |
|  | 1. Description of sample | What are the important characteristics of the sample? e.g. demographic data, date |  |
|  | 1. Interview guide | Were questions, prompts, guides provided by the authors? Was it pilot tested? |  |
|  | 1. Repeat interviews | Were repeat inter views carried out? If yes, how many? |  |
|  | 1. Audio/visual recording | Did the research use audio or visual recording to collect the data? |  |
|  | 1. Field notes | Were field notes made during and/or after the interview or focus group? |  |
|  | 1. Duration | What was the duration of the inter views or focus group? |  |
|  | 1. Data saturation | Was data saturation discussed? |  |
|  | 1. Transcripts returned | Were transcripts returned to participants for comment and/or corrrection |  |
| **Domain 3**  **Analysis and findings** | 1. Number of coders | How many data coders coded the data? |  |
|  | 1. Description of the coding tree | Did authors provide a description of the coding tree? |  |
|  | 1. Derivation of themes | Were themes identified in advance or derived from the data? |  |
|  | 1. Software | Derivation of themes |  |
|  | 1. Participant checking | Did participants provide feedback on the findings? |  |
|  | 1. Quotations presented | Were participant quotations presented to illustrate the themes/findings? |  |
|  | 1. Data and findings consistent | Was there consistency between the data presented and the findings? |  |
|  | 1. Clarity of major themes | Were major themes clearly presented in the findings? |  |
|  | 1. Clarity of minor themes | Is there a description of diverse cases or discussion of minor themes? |  |
